# The inflammatory response to simulated day and night emergency alarm mobilisations

**DOI:** 10.1371/journal.pone.0218732

**Published:** 2019-06-21

**Authors:** Jamie L. Tait, Brad Aisbett, Sarah J. Hall, Luana C. Main

**Affiliations:** 1 School of Exercise and Nutrition Sciences, Deakin University, Geelong, Australia; 2 Institute for Physical Activity and Nutrition (IPAN), School of Exercise and Nutrition Sciences, Deakin University, Geelong, Australia; Medical University Innsbruck, AUSTRIA

## Abstract

**Purpose:**

Responding to emergency alarms is a daily occurrence for personnel in safety-critical occupations, and is associated with negative health outcomes in this population. The purpose of the present study was to determine the acute inflammatory response to an isolated emergency alarm mobilisation in both day and night conditions.

**Methods:**

Sixteen healthy males (mean age 25 ± 4 years) spent four days and nights in a sleep laboratory and were required to mobilise to an emergency alarm either during the day (1558 h), or from nocturnal sleep (0358 h). Pro (TNF-α, IL-1β, IL-8, IL-6) and anti-inflammatory (IL-4 and IL-10) cytokine responses to each alarm mobilisation were compared to time-matched control conditions without the alarm and mobilisation stimulus.

**Results:**

Analysis revealed no significant drift of cytokine levels at 1400 h across the study (P≥0.139). The plasma concentration of anti-inflammatory cytokine IL-4 was 84% greater in the 2-h sampling period following night alarm mobilisation compared to a night control of gentle awakening (P = 0.049), no other condition-by-time interactions were observed. The majority of inflammatory concentrations did not significantly change between alarm mobilisation and control conditions, in either day or night trials.

**Conclusions:**

These findings may reflect the lack of a true emergency (and the perceived stress) for the alarm mobilisation, together with the neutralising effect of different circadian biorhythms on inflammatory cytokine concentrations.

## Introduction

Emergency alarms are ubiquitous in clinical environments, industry, and safety-critical occupations [1–3], with personnel exposed to alarms at any time across the day or night [4, 5]. In the context of emergency services (e.g. fire and paramedic services, the military and transport services), an ‘alarm mobilisation’ typically consists of an abrupt change from non-activity to brisk physical movement in response to an alarm, in preparing for departure to a critical event [1, 6]. Responding to an alarm in this manner has been associated with negative health outcomes in emergency service personnel, including adverse cardiovascular incidents [7]. In US firefighters, 13.4% of on-duty deaths attributed to Coronary Heart Disease (CHD) within a 10-year period, occurred during alarm mobilisation, while the odds of CHD death (e.g. sudden cardiac arrest) are 2.8 to 14.1 times higher during an alarm mobilisation, compared to during non-emergency tasks [7]. Furthermore, alarm mobilisation is also implicated in the elevated risk of cardiovascular disease (CVD)-related on-duty fatality for emergency service personnel compared to other occupations [8, 9], along with non-fatal CHD related events in firefighters [10]. Given this data, alarm mobilisation may trigger an acute physiological stress response which leads to adverse cardiovascular events in those with pre-existing CVD [8].

The exact mechanism for an elevated risk of CVD-related incidents during an alarm mobilisation is yet to be elucidated, however alarm response has been acutely shown to increase heart rate by up to 66 beats·min^-1^ [1, 5, 6, 11, 12], and stimulate the release of stress hormones such as adrenaline, noradrenaline and cortisol [11–13]. Firefighting itself places considerable strain on the cardiovascular system, with fire suppression activities reported to elevate heart rate to maximal levels that are sustained for hours, whilst altering blood flow [14, 15]. This repeated strain, in concert with the abrupt oscillations seen during an alarm mobilisation, may in part explain CVD-related death and injury. Another possibility is that alarm mobilisation may also trigger an inflammatory response that increases the risk of CVD-related incidents reported in firefighters. Associations between sympathetic and immune responses to acute psychological stress have been previously reported [16, 17], while elevated levels of inflammatory markers have been strongly linked with CVD [18, 19] and other adverse chronic health outcomes [20]. Meta-analytic studies have reported increased production of the inflammatory cytokines interleukin (IL)-6 and IL-1β across a wide variety of acute time-limited psychological stressors such as induced social stress or cognitive assessment tasks [21, 22]. However, no studies of acute, intense noise stress were included in these analyses, and notably, alarm mobilisation is typically much shorter in duration than the majority of these experimental stressors (≤ 90 s). Given these relationships, reported increases in heart rate following emergency alarm mobilisation during the day or night [11] may be accompanied by acute inflammatory responses. However, it is currently unknown if the psychological stress of alarms and the rapid movement of mobilisation can activate immune responses [21, 23].

Of further consideration is the potential for alarms to disrupt and restrict sleep in on-duty personnel who are required to respond to emergency alarms. It is unknown as to whether sleep disruption instigated by a night-time alarm mobilisation produces an acute inflammatory response. Moreover, mixed findings have been reported in the few published studies that have examined the acute response of cytokines after abrupt waking and disrupted night-time sleep in the second half of the night (e.g., awake from 0300–0600 h) [24, 25]. Strong evidence suggests that cytokine release exhibits circadian periodicity, with peak production and secretion of the pro-inflammatory cytokines; (i.e. TNF-α, IL-1β, IL-6 and IL-12) occurring during nocturnal sleep (i.e. from 0000-0500h) [26, 27–29]. This secretory pattern is thought to facilitate a bias towards cellular immunity, initiating adaptive immune processes in lymph nodes [30, 31]. This pattern is argued to be a corollary of negative entrainment by the diurnal periodicity of plasma cortisol, which is more than 1.5 times higher at 1600 h compared to 0400 h [30, 32, 33]. Conversely, greater concentrations of anti-inflammatory cytokines (e.g., interleukin (1L)-4 and IL-10) appear during daytime activity through the proliferation of T cells (T_h_2) [28], to foster immediate effector functions of humoral or specific immunity [31]. However if alarm mobilisation constitutes a psycho-physiological stressor which induces a degree of inflammation, and this is produced in every shift, it could have profound inflammatory and health implications over the course of a career in emergency services. Particularly when combined with the hormonal and cardiovascular escalations witnessed in acute settings [1, 3, 5, 6, 12].

Therefore, gaining an understanding of the acute inflammatory response to a single alarm is the first necessary step in elucidating any longer-term relationship between an alarm stress response and CVD-related mortality in emergency personnel [7, 9]. Furthermore, contextualising any responses in the afternoon (1600 h) and during the night (0400 h) accounts for circadian factors [26, 27]. As such, the aims of the present study were to compare acute pro and anti-inflammatory (tumor necrosis factor (TNF)-α, IL-1β, IL-8, IL-6, IL-4 and IL-10) responses to an isolated emergency alarm mobilisation during the day (1600 h), to a daytime control condition with no alarm and mobilisation, and to compare the inflammatory response following being woken to an alarm mobilisation at night (0400 h), to gentle awakening with no mobilisation. It was hypothesised that the pro-inflammatory response would be greater during each alarm mobilisation condition compared to their respective control conditions.

## Materials and methods

### Participants

Sixteen healthy male participants aged 25 ± 4 years (mean ± SD), body mass; 71.9 ± 7.7 kg, and body mass index; 23.1 ± 2.2 kg·m^-2^, were recruited for this study. Males were recruited as they make up the vast majority of all emergency service personnel (>97%; [34]), and may have different hormonal responses to women. Participants were screened prior to commencing the study, and were considered if they met inclusion criteria of: no existing medical conditions, non-smoker, no shift work or trans-meridian travel in the past month and not currently taking medication. This study was part of a larger project where heart rate and cortisol [11], sleep inertia outcomes [35], and sleep electroencephalography [36] have been published. Written informed consent was obtained prior to commencing the study, and all procedures were approved by the Deakin University Human Research Ethics Committee (Project no: 2012–338) and by the Central Queensland University Human Research Ethics Committee (Project no: EC00158).

### Study protocol

Participants spent four consecutive days and nights in the sleep laboratory. On the morning of Day 1, participants were familiarised with the blood sampling procedures. Baseline blood samples were collected at 1400 h on all days to test for any drift in baseline measures across the study. Participants were informed that that could receive an alarm at any time of the day or night on days 1, 3 or 4 and that only one alarm would be presented each 24 h. An emergency alarm stimulus from a 105-dB 15-W megaphone equipped with siren switch (ER-122155 TOA megaphone, Kobe, Japan), was presented outside the participants’ individual rooms at 1558 h on Day 1, for a maximum duration of 2 min. This alarm intensity is consistent with the literature and occupational practice [1, 6, 11–13, 37]. The 1558 h alarm time aligns with the period associated with the highest number of emergency alarms, and highest number of coronary heart disease related deaths in emergency service workers [8].Upon hearing the alarm, participants were required to don shoes and a protective jacket used in Australian firefighting agencies, and walk briskly to a testing station. Finger prick blood samples were collected after the mobilisation period at 1600 h (T0), and 15, 30, 45, 60, 90 and 120 min post-mobilisation, for later measurement of plasma cytokine concentrations. This sampling timeframe was selected to provide a comprehensive picture of the cytokine response [21], as it is hypothesised that the typical re-appearance of cytokines at 45 min post-stressor is attributed to their re-release into the bloodstream after a migration to target tissues, or a delayed expression of cytokines [21]. Time-matched samples were collected on Day 2 for the daytime control condition, where no alarm was presented and participants were requested to slowly make their way to the testing room ≃10 min before T0.

No alarms were presented during the afternoon in either Day 3 or 4, despite participants expecting an alarm at any time of the day. On the third night, participants received one of two conditions at 0358 h, an alarm stimulus requiring mobilisation to the testing station (alarm condition), or a gentle awakening (control) condition. This time was chosen to contextualise the circadian nature of cytokines. In the night alarm condition, an alarm was presented outside participant’s rooms, as per the day alarm condition. Upon alarm sounding, lights were brought up to their full level, and participants then followed the same protocol as the day alarm mobilisation. Participants were exposed to the alternate condition on the fourth night. In the night control condition incorporating gentle awakening, lights in participant rooms were activated externally but only brought up to a half level, after which researchers quietly knocked and entered to provide verbal commands. Participants remained in their beds but slowly sat up to provide the T0 finger prick blood sample. Following this they walked slowly to desks positioned in their rooms to provide all other samples. The same collection time points were used for sampling in both day and night conditions. In both night-time test conditions, subjects did not return to sleep after data collection (0400–0600 h), and therefore a cross-over design was employed (n = 8 for night alarm then night control; n = 8 for night control then night alarm) to counterbalance the potential pro- or anti-inflammatory effects of sleep restriction.

Dietary intake was controlled throughout the study, and no food was consumed during the 90 min prior to blood collection. In an attempt to further regulate emotion, alertness and stress levels, no caffeinated beverages were allowed for the duration of the study. Similarly, participants spent the majority of their time in individual bed-sit style rooms, and did not have access to natural light for the duration of the study due to the design of the sleep laboratory. Physical exertion was not permitted, however participants were encouraged to perform light stretching if they reported joint or muscle stiffness.

### Blood sampling and cytokine analysis

Finger prick blood samples were collected using BD-microtainer retractable lancets (Becton-Dickinson, Franklin Lakes, NJ). Samples (≥375 μL) were collected in a 500-μL micro-test-tube coated with K_2_EDTA (Becton-Dickinson, Franklin Lakes, NJ), and immediately stored on ice until the end of the sampling period. Samples were then centrifuged at 5,000 rev·min^-1^ (Sigma 203, Sigma, Osterode am Harz, Germany) for 10 min to separate plasma, and stored at -80°C until analysis. Finger prick blood sampling was chosen to avoid the blood coagulation effects, and immune challenges produced in samples obtained via venupuncture and intravenous cannulation [38].

Inflammatory cytokines IL-4, IL-6, IL-1β, TNF-α, IL-10 and IL-8 were analysed simultaneously using a 6-plex Milliplex^TM^ MAP Human High-Sensitivity Cytokine kit (Cat. No: HSCYTMAG-60SK; Millipore Corp, St Charles, Missouri). Each assay was performed strictly according to the manufacturer’s protocol for plasma samples. The mean inter-assay coefficient of variation was 9.5% and the mean intra-assay coefficient of variation was 9.3%.

### Statistical analyses

Due to the larger aims of the project, and a lack of research regarding inflammatory response to an alarm, a priori power calculations were based on changes in heart rate in response to an emergency alarm [1]. From this study we conservatively adopted the mean and the mid-point of the reported range of heart responses, as none of the studies in the alarm literature reported standard deviations. This power analysis indicated that a sample size of 14 was required. Further, because of the absence of suitable data to generate power analyses for other stress markers of interest, the proposed sample size (n = 16) was consistent with stressor-related firefighter research [39, 40].

Statistical analyses for cytokine samples were completed using STATA statistical software release 15.0 (STATA, College Station, TX, USA). Cytokine data were assessed for normality using the Kolmogorov-Smirnov Test which is suitable for small samples [41]. Due to non-normality, inflammatory biomarkers were log transformed prior to analysis. The response of inflammatory cytokines between and within conditions was analysed using General Linear Mixed Models (GLMM) with random effects, adjusting for the variability between clusters (day of testing, which equates to order of testing) and within a cluster (participants). The condition (alarm or control) was the fixed effect, and clusters (day of testing) and the unit of analysis (participants), were included as random effects to account for the clustered design. This reporting method attempted to eliminate inter-plate bias and individual variability between baseline (1400 h) and experimental concentrations. GLMMs also account for the possibility of autocorrelation in the repeated cytokine measurements (i.e., samples and/or days) on each individual, or serial correlation of data points over time, by including a model for the covariance structure, and are increasingly preferred over repeated-measures ANOVA for these types of data [42]. Samples that were below the lowest detectable limit were assigned the lowest detectable concentration for their assay. From a potential 512 samples for each cytokine, 23 samples could not be obtained for IL-4, IL-1β and TNF-α analyses, and 3–7% of all samples were excluded from analyses as they were >15 standard deviations above the mean. For descriptive purposes, within- and between-condition changes are expressed as percentages change, which represent the absolute difference from T0 in the log-transformed data multiplied by 100 [43]. Within-condition differences were calculated by subtracting the changes from T0 for each sample. Between-condition differences were calculated by subtracting the within-condition changes from T0 for the alarm condition, from the within-condition changes from T0 for the control group [43]. Concentrations at baseline were also compared to T0 to emphasise any circadian patterning of inflammatory cytokines, compared to experimental conditions. All data are presented as means ± SEM (standard error of the mean), and the significance level was set at P<0.05.

## Results

### Day alarm inflammatory cytokine response

**Fig 1:**
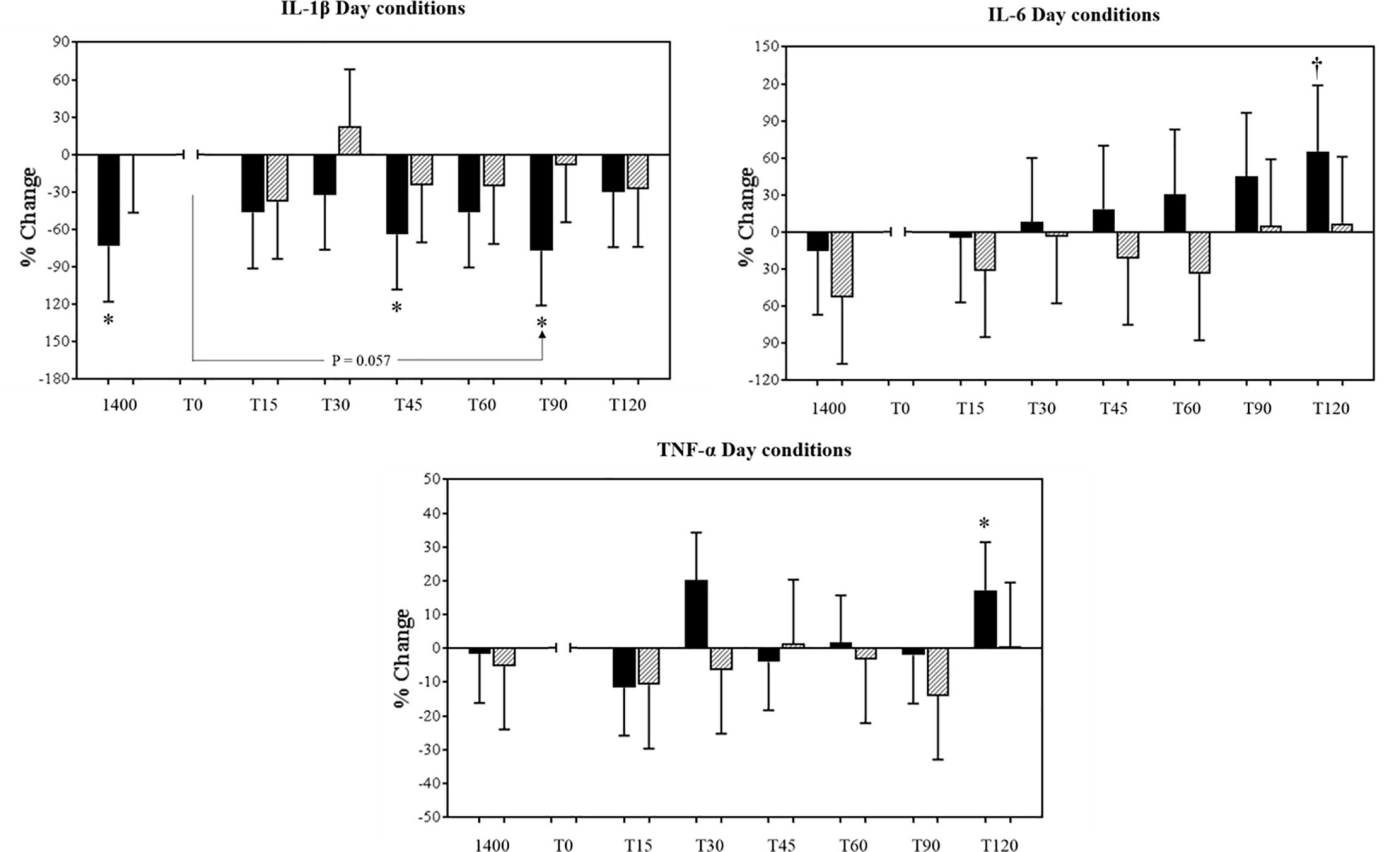
Mean (± SEM) percentage changes in Interleuklin-1 beta (IL-1β), Interleuklin-6 (IL-6) and tumor necrosis factor alpha (TNF-α) log-transformed plasma concentrations during 2-h of physiological sampling following a day alarm mobilisation, relative to T0 for each condition, time matched to control conditions. Dark bars (day alarm), light bars (day control). * P<0.05, † P≤0.001 within-condition change relative to T0, and between-condition changes relative to T0 (denoted by line and P-value).

The mean baseline plasma cytokine concentrations collected at 1400 h on the day of each condition are shown in Table 1, along with mean concentrations at T0 for each condition. Analysis revealed no significant drift of cytokine levels at 1400 h across the study, and no differences in these values between conditions (P≥0.139). Comparing the day conditions, there was a trend for a significant 68% net decrease (between-condition difference for the change over time) in IL-1β at T90 compared to T0 in day alarm condition, compared to day control (condition-by-time interaction P = 0.057; Fig 1).

**Table 1:** Mean inflammatory marker values at 1400 h (baseline) and time of alarm onset (T0) for each condition.

Biomarker	Day alarm		Day control		Night alarm		Night inactive	
	Baseline	T0	Baseline	T0	Baseline	T0	Baseline	T0
*Pro-inflammatory*								
IL-6, pg/mL	7.78 ± 5.36	7.81 ± 4.93	4.00 ± 1.85	7.97 ± 3.81	3.74 ± 1.27	4.05 ± 1.53	7.77 ± 6.25	6.67 ± 4.21
TNF-α, pg/mL	5.52 ± 0.42	5.54 ± 0.34	8.27 ± 2.07	7.32 ± 1.08	5.16 ± 0.51	6.99 ± 1.29	4.86 ± 0.66**	6.23 ± 1.22
IL-1β, pg/mL	4.06 ± 0.78*	7.10 ± 1.30	6.76 ± 1.98	6.30 ± 1.60	7.43 ± 1.97	7.19 ± 1.56	4.97 ± 0.86	4.60 ± 0.97
IL-8, pg/mL	3.88 ± 0.29	5.55 ± 1.37	4.61 ± 0.73	7.49 ± 1.82	4.45 ± 0.57**	8.63 ± 2.25	4.08 ± 0.68	4.80 ± 0.89
*Anti-inflammatory*								
IL-10, pg/mL	44.2 ± 20.0	40.4 ± 16.0	37.7 ± 22.1	52.04 ± 22.2	36.7 ± 12.6	53.0 ± 20.9	25.99 ± 9.34	21.6 ± 6.49
IL-4, pg/mL	32.1 ± 10.3	34.3 ± 10.8	28.2 ± 9.10	44.7 ± 15.7	35.6 ± 10.7*	26.9 ± 8.37	21.9 ± 6.89	24.5 ± 7.14

Baseline values are means ± SEM.

* P<0.05

** P<0.010 relative to T0.

This was due to a 77% decrease in concentration at T90 compared to T0 in the day alarm condition (P = 0.014), and a 9% decrease in the day control condition that did not reach statistical significance (P = 0.813; Fig 1). For all other inflammatory measures in day conditions there were no significant interaction effects, or main effects for condition (P>0.065). In the day alarm condition, within-condition changes were detected; concentrations of IL-6 were 65% higher and concentrations of TNF-α were 17% higher at T120 compared to their values at T0 (P = 0.018 and P = 0.001 respectively; Fig 1), indicating an increase of these pro-inflammatory cytokines over the sampling time. Furthermore, a 73% increase in the concentration of IL-1β at T0 compared to 1400 h (P = 0.040), and a 64% decrease in IL-1β at T45 compared to T0 (P = 0.040) were also observed.

### Night alarm inflammatory cytokine response

**Fig 2:**
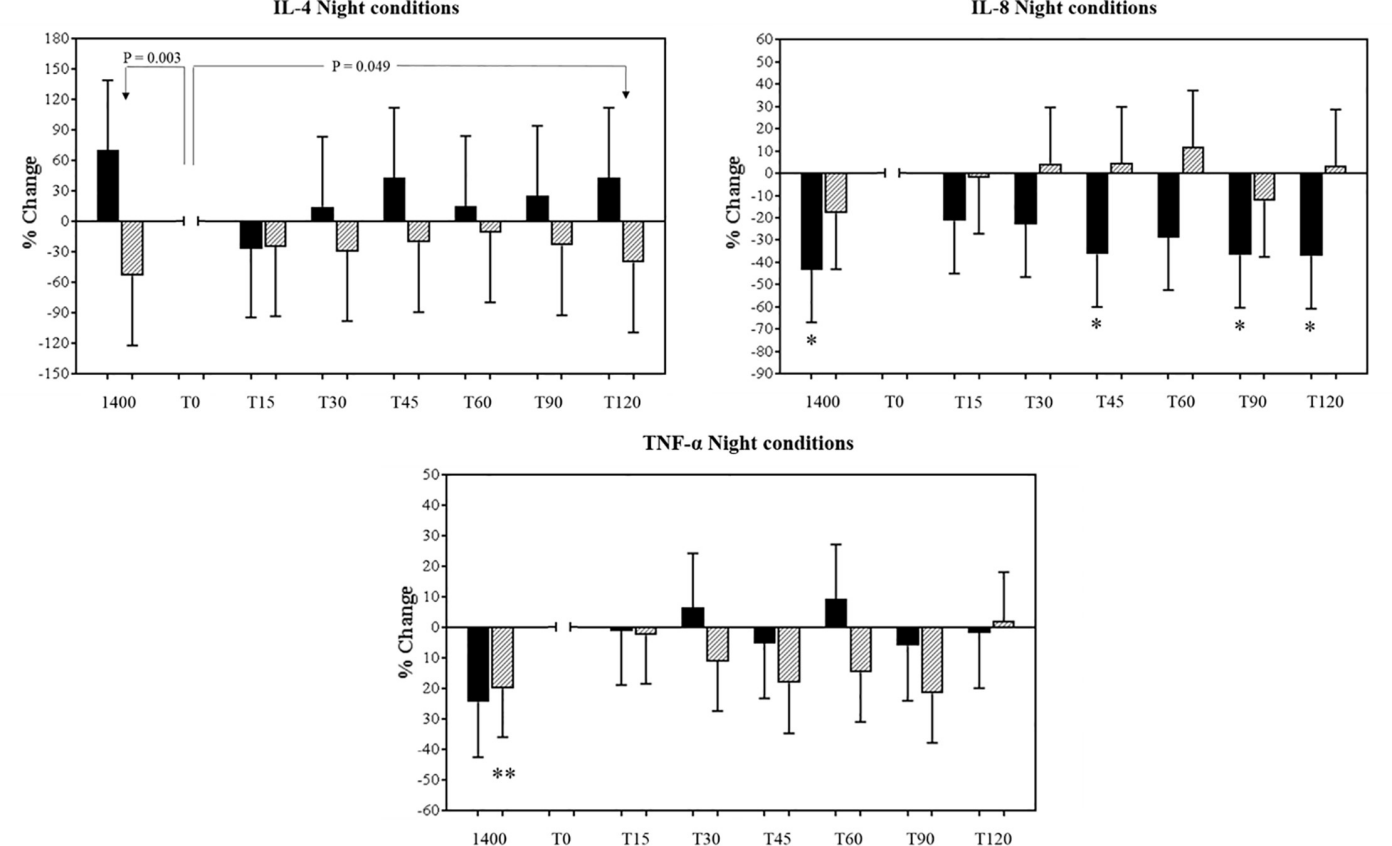
Mean (± SEM) percentage changes in Interleuklin-8 (IL-8), Interleuklin-4 (IL-4) and tumor necrosis factor alpha (TNF-α) log-transformed plasma concentrations during 2-h of physiological sampling following a night alarm mobilisation, relative to T0 for each condition, time matched to control conditions. Dark bars (night alarm), light bars (night inactive). * P<0.05, ** P<0.010 within-condition change relative to T0, and between-condition changes relative to T0 (denoted by line and P-value).

Comparing the night conditions, there was a significant 84% net increase (between-condition difference for the change over time) in IL-4 at T120 compared to T0 in the night alarm condition, compared to night inactive (condition-by-time interaction P = 0.049). This was due to a 43% increase in concentration at T120 compared to T0 in the night alarm condition (P = 0.168), and a 41% decrease in the night inactive condition (P = 0.165; Fig 2), with neither of these changes reaching statistical significance. There was also a 17% (between-condition difference for the change over time) net decrease in IL-4 concentration at T0 compared to 1400 h in the night alarm condition compared to night inactive (condition-by-time interaction P = 0.003). This was due to a significant 70% decrease in concentration at T0 compared to 1400 h in the night alarm condition (P = 0.018), whereas in the night inactive condition, concentrations at T0 showed a tendency to be higher compared to 1400 h (53%, P = 0.068). For all other inflammatory measures in night conditions there were no significant interaction effects, or main effects for condition (P>0.099).

There were no other significant within-group changes with the exception that after T0, there was a tendency for concentrations of IL-8 to decrease across the sampling period in the night alarm condition. Specifically, concentrations at T0 were; 36% higher than at T45 (P = 0.021), 37% higher than T90 (P = 0.020), 37% higher than T120 (P = 0.036), and 43% higher than baseline levels taken at 1400 h (Fig 2). Further, concentrations of TNF-α were 20% higher at T0 than the 1400 h sample in the night inactive condition (P = 0.006; Fig 2).

## Discussion

The overarching aims of this study were to determine whether day and night alarm mobilisations could be classified as acute immunological stressors. Changes in the plasma concentration of anti-inflammatory cytokine IL-4 were higher in the 2-h sampling period following night alarm mobilisation compared to a gentle awakening control condition (P = 0.049), however no between-group differences over time were seen for any cytokine following day alarm mobilization, compared to the day control condition. In light of these findings, it is unclear whether a single alarm mobilisation can be classified as an acute immunological stressor.

### Associations between inflammatory response and sympatho-adrenal medullary activation

A relationship between sympathetic activation and immune response has been previously reported, with studies demonstrating correlations between acute changes in heart rate and inflammatory markers [17, 44]. Furthermore, significant increases in heart rate have been reported after both day and night alarm mobilisation, compared to control conditions, in healthy males [11]. Despite these findings, the response of the pro-inflammatory markers IL-6, IL-8, TNF-α and IL-1β following alarm mobilisation were no different to their control conditions. Activation of the sympatho-adrenal medullary (SAM) system has been previously associated with the release of catecholamines which lead to the expression of pro-inflammatory genes and products by immune cells [45], particularly in response to acute stress [46]. For example, Steptoe et al. [17] employed stress tasks of 10 min total duration, revealing significant positive associations of cytokines with heart rate and other sympathetic measures under stress conditions (TNF-α and heart rate 2-h post task; *r* = 0.66, P = 0.028). The current data therefore implies that an alarm mobilisation in a non-emergency situation, using ‘healthy normal’ participants, may not be capable of provoking a pro-inflammatory response, despite its ability to activate the SAM system [11].

To our knowledge, there are no previous studies that have evaluated changes in circulating inflammatory markers following alarm mobilisation. In the current study, there are a number of factors which may explain why we observed no between-condition changes in the various inflammatory markers relative to control conditions, including an inadequate exposure to the psychological stressor, and the nature of the stressor used in our protocol. An initial explanation for our lack of between-condition change in the pro-inflammatory markers in day or night conditions relates to controllability, in that the laboratory-based alarm stimulus was not perceived by the participant to be a serious stressor and subsequently failed to elevate inflammatory levels [47]. Studies have provided evidence that situational determinants such as controllability over a stressor [48], the challenge of a task to participant self-presentation [17], and emotion and mood states [49, 50], may influence the nature and magnitude of the immune response induced by acute stress. This data was not collected in the current study, however it is reasonable to speculate that the participants had high degree of control and low perception of stress due to the simulated setting. Higher control expectancy and a lower perception of the stressfulness of an external stressor have been previously suggested to act as protective factors for stress-induced inflammation [48]. Further studies including emergency service personnel may reveal different immunological responses to a stimulus that has an element of realism with the potential to psychologically impact the worker. Secondly, the present protocol included an alarm stimulus and response that lasted for a maximum of 2 min, which although is occupationally relevant (i.e. need to depart station within 90s), may have been insufficient to elicit a response. Previous studies have reported significantly higher levels of inflammation after exposure to stressors longer than 5 min [17, 21, 22], with the majority of these studies not employing a comparative control condition. Although the alarm stimulus was not shown to be associated with inflammatory response in a non-emergency simulation, the recreation of other working conditions with longer alarm stimuli (e.g., engineers, paramedics), or repeated alarms, may yet produce detrimental physiological responses.

### Pro-inflammatory conditions during day alarm mobilisation

It is established that acute laboratory stress is associated with an increase in circulating and stimulated concentrations of pro-inflammatory biomarkers [21, 22], but we did not observe any differences between the alarm and control condition. However, within the day alarm condition, concentrations of IL-1β exhibited a 73% increase immediately after alarm mobilisation (T0), compared to baseline levels collected two hours prior (1400 h), after which there was a tendency for IL-1β concentrations to decrease over the remaining 90 minutes testing period. This data contrasts with daytime research which has reported an increase of this biomarker following acute exposure to psychological stress tasks [21, 22], or immunological challenge [47], and may relate to the factors discussed above. As IL-1β operates primarily at the paracrine level, increased production of this cytokine may be more apparent following white blood cell stimulation in-vitro [21]. In addition, the lack of between-condition change in TNF-α, IL-1β, IL-6 and IL-8 concentrations daytime conditions may reflect the circadian pro-inflammatory downregulation at 1400 h and 1600 h (T0; [26, 27, 28]). The regulatory measures of anti-inflammatory cytokines during this daytime period [31, 51] may therefore be a more powerful influence on immune response than any alarm mobilisation stimulus.

In contrast to our findings for IL-1β, concentrations of IL-6 and TNF-α demonstrated significant increases two hours after day alarm mobilisation (65% and 17% respectively), compared to their relative concentrations at alarm onset (T0). However, these were not different to concentrations in the control condition. Meta-analytic data has previously reported increases of IL-6 and TNF-α in response to a range of acute psychosocial stress tasks and naturalistic stressors, typically between 45–120 min after exposure to the stressor [21, 22]. In particular, moderate-large increases of IL-6 appear at 90 and 120 min following stress exposure [52], with these response patterns thought to reflect the half-lives of these cytokines, or differences in clearance mechanisms [53]. For example, Steptoe et al. [17] reported increased concentrations of IL-6 (56%; P<0.05) two hours after the completion of acute mental tasks. The responses of TNF-α to a psychological stressor have been less consistent, reflecting considerable heterogeneity across studies in terms of the different populations and analytic methods used [52].

Pertinent to emergency service personnel, elevated levels of circulating IL-6 have been linked to the increased synthesis of C-reactive protein, with both of these markers implicated as predictors of myocardial infarction and arterial disease in healthy men [54, 55]. Although acute increases in IL-6 reflect a normal physiological response to stress, alarm mobilisations routinely performed over an emergency service career could facilitate a chronic low-grade inflammatory response. A prolonged and accumulated exposure to these dysfunctions may therefore contribute to the progression of CVD through enhanced blood pressure and heart rate, atherosclerotic plaque formation and cardiac irritability [19, 56]. Therefore, an increase in IL-6 in the day alarm condition cannot be currently linked with CVD-related incidents, however IL-6 could yet be an important biomarker for the association with CVD.

### Inflammatory conditions during night alarm mobilisation

At the onset of night alarm mobilisation, concentrations of the anti-inflammatory cytokine IL-4 underwent a significantly greater decrease from baseline, compared to the night control condition (Fig 1), before experiencing a significantly greater increase over the 2-h sampling period compared to gentle awakening. Given the dearth of acute stress studies involving anti-inflammatory cytokines, especially at night, it is unclear what the relevance of this increase is given the other inflammatory cytokines did not significantly change during the night alarm mobilisation. Our findings partially support previous meta-analytic findings that circulating levels of anti-inflammatory cytokines may transiently decrease in the initial stages of a stressor, before showing small increases over a 90 min period [52]. However other evidence suggests that the anti-inflammatory response to a stressor may be context specific. For instance, significantly lower levels of stimulated IL-4 and IL-10 were witnessed in high-anxiety students measured one day before an academic examination [50], while a videotaped speech task stressor did not affect IL-4 levels [57]. Despite this collective, but limited data, few of these studies included a control condition, and all were conducted during the day, when anti-inflammatory cytokines are at their diurnal peak [28, 31]. Compared to these studies, our use of distinct within-participant control conditions counteracts the individual variability of circulating cytokine concentrations, and delineates cytokine responses in different phases of their circadian cycles [26, 28].

Significant elevations of the anti-inflammatory cytokine IL-4 have been previously witnessed as a result of surgical stress [58], which were suggested to indicate a shift in the balance toward antibody-mediated immunity due to stress-related glucocorticoid release [58]. Furthermore, positive associations between anti-inflammatory cytokines and glucocorticoids exist [59, 60], and therefore it could be hypothesised that the gradual rise of IL-4 during the night alarm condition may be associated with a rise in cortisol following an alarm mobilisation [11], and/or the cortisol awakening response [61]. Further, dramatic increases in this hormone and serum adrenocorticotrophin have been associated with abrupt and forced awakening occurring four hours earlier than expected [33, 62]. Therefore, due to a lack of supportive evidence, the clinical significance of this increase in IL-4 cannot be determined. However, an increase in IL-4 may act as a protective countermeasure to any rise in pro-inflammatory cytokines at alarm onset [50, 59]. Moreover, IL-4 may transiently respond to an unknown aspect of a nocturnal alarm mobilisation, which may modulate the release of pro-inflammatory cytokines across the 2-h sampling period, which may explain why only an initial increase in the cytokine IL-8 was detected following night alarm mobilisation, compared to its own afternoon levels.

Contrary to our hypothesis, pro-inflammatory cytokine concentrations did not increase following a night alarm mobilisation compared to the control condition. There is evidence that peak production and secretion of the pro-inflammatory cytokines occurs during nocturnal sleep (0000–0500 h) [27, 29], and therefore an initial explanation may be that the high expression of these biomarkers during our night sampling period may have obscured any stress-related rises. Second, regulatory interactions have been consistently reported between hypothalamic-pituitary-adrenal system products and immune responses [59, 63]. Our combination of alarm mobilisation [11], and the forced awakening [33, 62], may have led to augmented cortisol concentrations which dampened pro-inflammatory cytokine expression in the night alarm condition [63, 64]. This may partially explain the within-condition decreases in IL-8 following night alarm mobilisation, while forced awakening may have also modulated inflammatory levels in the control condition. Further studies examining cortisol and immune measures are required.

There were several strengths to this study, most notably it is the first trial to investigate the inflammatory response to an acute alarm mobilisation, which may be viewed in part as a stressful auditory and physical stressor, during periods of day and night. Furthermore, the use of within-participant control conditions attempted to counteract the individual and circadian variability of circulating cytokine concentrations [26, 28]. Future occupational research designs may investigate whether a single or repeated alarm mobilisation at another point(s) in the circadian cycle provokes an immune response. The findings of this study may have been subject to potential limitations. Firstly, the order of day conditions was fixed throughout the study, with alarm mobilisation on Day 1 and day control on Day 2. However, a cross-over in both day conditions and night conditions may have led to a sequence of repeated alarms, which was not the focus of the study. In addition, the commencement of each condition on consecutive days was designed to minimise unnecessary participant burden incurred by washout periods, and may reflect authentic on-shift experiences, as alarms are expected at all times but do not always occur. However this continual succession may have had some influence on immune responses. Second, the confinement of participants in a controlled environment was necessary to regulate emotion and mood, however this environment may not be representative of a real world station-house or barracks. Furthermore, the recruitment of healthy norms was a practical choice for proof of concept, and was necessary to isolate the physiological and immune alarm responses from the underlying physiology of emergency workers [9]. Further research on alarm mobilisations should undoubtedly include emergency service personnel to elucidate whether a response may be less stressful because it can be curtailed by a rational representation (i.e. “it’s just part of the job”), or exacerbated by a more traumatic significance to emergency service personnel [34]. Third, it is unclear whether the lancet puncturing we employed creates localized immune challenges, or if any apprehension of impending finger pricks may have influenced immune response. Increases in IL-6 across a 24-h period have been attributed to local changes brought about by intravenous sample collection [65], and repeated finger prick blood sampling may also influence inflammatory release. Lastly, we employed linear mixed models in our analyses as we had relatively few temporal measurements according to our study design. This represents a potential limitation as serial dependencies in temporal data are not only due to circadian rhythms but also other regular influences on the data measured (everyday activities, emotions etc.). To deal with these regular serial influences more appropriately, future research would require more frequent psychobiological stress measurements per participant (i.e. at least 20–50 consecutive measurements depending on the modelling technique), which would then allow the modelling and analysis of time-series data, with subsequent analysis by generalized estimating equations to generalise the findings. This method would account for any intra-individual serial dependencies created through the interaction between immune responses and external influences (e.g., circadian rhythms, emotion) across the testing period, and potentially capture further alarm-immune relationships.

In summary, a night alarm mobilisation was associated with greater increases in IL-4 concentration across a 2-h sampling period, in comparison to gentle awakening. However, a day alarm mobilisation did not produce an inflammatory response in comparison to a relative control condition. No other significant between-condition changes over time were observed for any other cytokine, and there was no drift of cytokine levels at 1400 h across the study. The current findings suggest a lack of response of inflammatory markers to alarm mobilisation, potentially mediated by circadian, physiological and psychological mechanisms. Currently, it cannot be determined whether an alarm mobilisation *per se* may constitute a definitive immunological stressor. Future research may consider whether an acute inflammatory response may be elicited with repeated alarms, alarm mobilisations presented at different times during a circadian cycle or in actual emergency conditions. With this information, any injurious properties of habitual alarm mobilisation may be better understood.
